# Heterogeneous genetic and non‐genetic mechanisms contribute to response and resistance to azacitidine monotherapy

**DOI:** 10.1002/jha2.527

**Published:** 2022-07-08

**Authors:** Vasiliki Symeonidou, Marlen Metzner, Batchimeg Usukhbayar, Aimee E. Jackson, Sonia Fox, Charles F. Craddock, Paresh Vyas

**Affiliations:** ^1^ MRC Molecular Haematology Unit, Oxford Biomedical Research Centre Haematology Theme, Oxford Centre for Haematology, Weatherall Institute of Molecular Medicine, Radcliffe Department of Medicine University of Oxford Oxford UK; ^2^ Cancer Research UK Clinical Trials Unit University of Birmingham Birmingham UK; ^3^ Centre for Clinical Haematology Queen Elizabeth Hospital Birmingham UK

**Keywords:** acute myeloid leukaemia, azacitidine, clonal evolution, hypomethylating agents, immune microenvironment

## Abstract

Acute myeloid leukaemia is prevalent in older patients that are often ineligible for intensive chemotherapy and treatment options remain limited with azacitidine being at the forefront. Azacitidine has been used in the clinic for decades, however, we still lack a complete understanding of the mechanisms by which the drug exerts its anti‐tumour effect. To gain insight into the mechanism of action, we defined the mutational profile of sequential samples of patients treated with azacitidine. We did not identify any mutations that could predict response and observed lack of a uniform pattern of clonal evolution. Focusing on responders, at remission, we observed three types of response: (1) an almost complete elimination of mutations (33%), (2) no change (17%), and (3) change with no discernible pattern (50%). Heterogeneous patterns were also observed at relapse, with no clonal evolution between remission and relapse in some patients. Lack of clonal evolution suggests that non‐genetic mechanisms might be involved. Towards understanding such mechanisms, we investigated the immune microenvironment in a number of patients and we observed lack of a uniform response following therapy. We identified a higher frequency of cytotoxic T cells in responders and higher frequency of naïve helper T cells in non‐responders.

## 1 INTRODUCTION

Acute myeloid leukaemia (AML) is an aggressive and clinically heterogenous blood cancer that is most commonly diagnosed in patients over the age of 65 years [1]. Intensive induction chemotherapy with or without allogeneic haematopoietic cell transplantation (HCT) is the standard treatment for AML [2]. However, older unfit patients are often deemed ineligible for intensive chemotherapy due to the high risk of treatment‐related mortality (TRM) [2]. For these patients, treatment alternatives remain limited with hypomethylating agents such as azacitidine being at the forefront [3, 4, 5, 6, 7]. Following therapy with azacitidine there is an increase in the incidence of remission and overall survival of these patients [3, 5, 6, 8, 9]. However, it should be noted that such therapy is non‐curative and response is only temporary as all patients relapse. Additionally, not all patients will initially respond to therapy and we currently have no biomarkers that could predict response to azacitidine. Our inability to predict response stems from our lack of understanding of the exact mechanism by which the drug exerts its clinical anti‐tumor effect. The aim of this study was to investigate genetic and non‐genetic mechanisms that might play a role in response and resistance to azacitidine monotherapy.

## 2 MATERIALS AND METHODS

### 2.1 Patient samples

Bone marrow (BM) and peripheral blood (PB) samples from AML and MDS patients were obtained with informed consent and collected by research ethics committee approved Biobanks (described in detail in Craddock et al) [10]. Mononuclear cells (MNCs) were isolated by Ficoll density gradient and MNCs were viably frozen in 90% FCS with 10% DMSO in liquid nitrogen.

Additional materials and methods are in the supplementary materials and methods.

## 3 RESULTS

### 3.1 Mutational profile is not a predictor of response to azacitidine monotherapy

**FIGURE 1:**
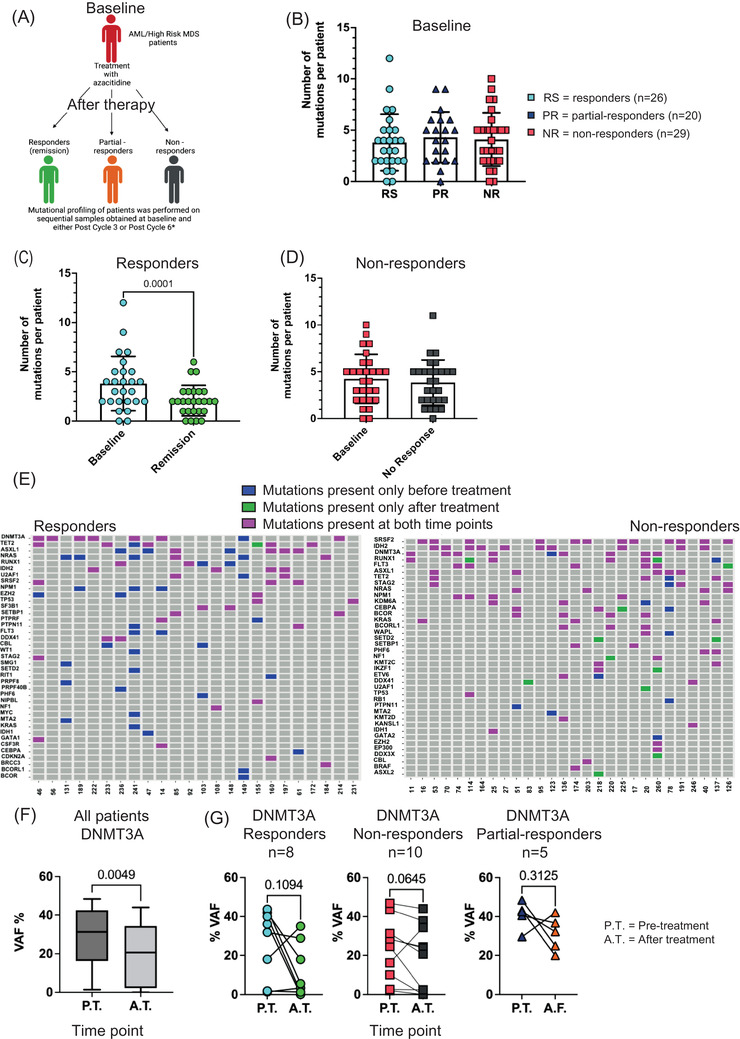
Delineation of the mutational profile of patients before and after azacitidine treatment. (A) Schematic of experiment plan. ‘Created with BioRender.com’. *For some patients the after therapy time point is other than PC3 or PC6, and it is clearly indicated in Table S3. (B) Number of mutations in responders (RS), partial‐responders (PR) and non‐responders (NR) at baseline. Analysis of variance (ANOVA) test was performed. (C) Number of mutations in responders at baseline and remission. Each point represents a patient. Wilcoxon matched pairs signed rank test was performed. (D) Number of mutations in non‐responders at baseline and no response. Each point represents a patient. Wilcoxon matched pairs signed rank test was performed. (E) Mutations present before and after azacitidine treatment in responders and non‐responders. Blue = mutations present only before treatment, green = mutations present only after treatment, magenta = mutations present before and after treatment. The y‐axis shows the genes, where the mutation is present and the x‐axis the individual patient. (F) Variant allele frequency (VAF) of *DNMT3A* mutations in all patients pre‐treatment (PT) and after treatment (AT) (*n* = 23). Wilcoxon matched pairs signed rank test was performed. (G) VAF of *DNMT3A* mutations in all groups of patients pre‐treatment (PT) and after treatment (AT). Each point represents a patient, and each line connects the two time points. Wilcoxon matched pairs signed rank test was performed

AML is a genetically complex disease characterised by clonal heterogeneity driven by acquired somatic mutations. To study the clonal basis of response and resistance to azacitidine therapy we performed targeted sequencing using a custom‐made panel that allowed detection of somatic variants of 108 genes commonly mutated in myeloid malignancies (Table S1). This was performed in sequential samples obtained from 75 patients (Table S2) that were previously described in Craddock et al. and were part of the RAvVA trial (ISRCTN68224706, EudraCT 2011‐005207‐32) [10]. The sequential samples included, an initial sample obtained at baseline and a second sample obtained after therapy and in particularly either post cycle 3 (PC3) or post cycle 6 (PC6) (Figure 1A and Table S3). For a few patients the after therapy sample was obtained at PC5 or PC12 (Table S3). It should be noted that the after therapy sample was obtained when the patient achieved remission (for responders), partial response (for partial‐responders) and no response (for non‐responders). Additionally, where available we sequenced samples obtained at a later time point (pre‐relapse) or at relapse. Following targeted sequencing of the aforementioned samples, the median coverage was 750X and we observed consistent coverage across all genes (Figure S1A,B). We identified at least 1 mutation in 93% of patients (number of mutations ranging from 1–12) (Table S3). We divided our cohort into 3 groups based on the clinical response or lack thereof. Initially, we observed that the number of mutations at baseline is not a predictor of response as the 3 groups shared a similar number of mutations (Figure 1B and Table 1).

**TABLE 1:** Number of patients included in this study and average number of mutations per group

Clinical response	Number of patients	Average number of mutations at baseline
Responders	26	3.8
Partial‐responders	20	4.3
Non‐responders	29	4.1

To elucidate if there are changes in the mutational landscape following azacitidine therapy, we examined changes in the number of mutations per group before and after treatment. Interestingly, following therapy the number of mutations decreased only in patients that responded (RS) to therapy at remission, but not at relapse (RL) (Figure 1C and Figure S1C). We did not observe any changes in non‐responders and patrial‐responders (Figure 1D and Figure S1D,E). A closer look into the mutations that persisted following therapy (at remission) in responders revealed that they were in genes associated with pre‐leukaemia (*ASXL1*, *DNMT3A*, *IDH2, SRSF2* and *TET2*) [11, 12, 13, 14, 15, 16] (Figure 1E). In patients with refractory disease and partial response to azacitidine monotherapy, we observed that the majority of mutations persisted after therapy (Figure 1E and Figure S1F). In a small number of patients, some mutations were eliminated whereas others were acquired. Importantly, there was no clear pattern of elimination or acquisition of these mutations.

In addition to individual mutations, we wanted to investigate if co‐occurrence of mutations could be a predictor of response. To visualise the presence of co‐occurring mutations at baseline and at remission, we utilised the circos plots (Figure S1G,H). Focusing on patients that responded to therapy, we did not identify any pair of mutations to be more prominent. This is visible from the circos plots as all connecting ribbons have similar width. Additionally, we observed a decrease in the mutational complexity of the disease at remission compared to the baseline. This is visible from the figures as there is a reduced number of co‐occurring mutations following therapy. We did not observe any changes in non‐responders (data not shown).

To investigate the effect that azacitidine exerts on specific mutations, we compared the variant allele frequency (VAF) of the 5 most frequent mutations (*DNMT3A*, *TET2*, *ASXL1*, *SRSF2*, *IDH2*) before and after therapy in all patients. Interestingly, we observed that the VAF of *DNMT3A* mutations decreased following therapy in all 3 groups of patients (Figure 1F,G).

From these data we conclude that there is no correlation between a specific mutational signature and response or primary resistance to azacitidine therapy. We observed an overall decrease in the mutational burden following therapy only in responders. Interestingly, we observed that pre‐leukemic mutations persisted after therapy and there was a modest but uniform decrease in the VAF of *DNMT3A* mutations.

### 3.2 Clonal basis of primary resistance to azacitidine monotherapy

Defining the mutational landscape in patients’ samples is crucial in molecularly profiling the disease. However, understanding clonal evolution is equally important. Therefore, having initially identified the mutations present before and after azacitidine therapy, we next wanted to investigate the clonal basis of response, primary and secondary resistance (relapse). Towards this end we investigated the variant allele frequency (VAF) of each mutation detected at the different time points.

**FIGURE 2:**
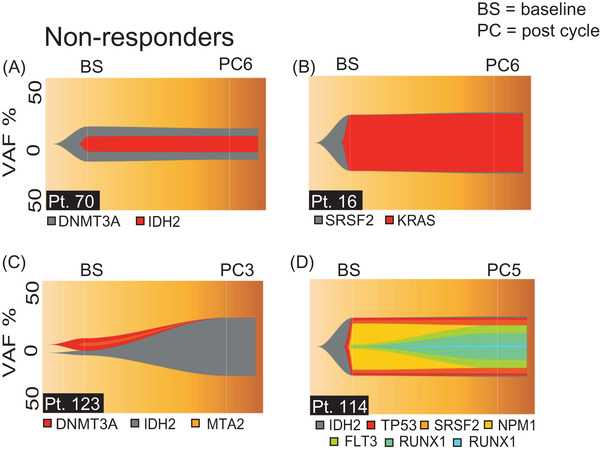
Clonal basis of primary resistance to azacitidine monotherapy. (A–D) FISH plots of patients with refractory disease, the two time points depicted in the FISH plots represent mutations and VAFs detected following NGS of samples obtained at baseline (BS) and after therapy. After therapy, time points are indicated in the figure. PC = post cycle (A) FISH plot of patient 70, (B) FISH plot of patient 16, (C) FISH plot of patient 123, (D) FISH plots of patient 114

We initially focused on patients that did not respond to therapy in order to investigate the clonal basis of primary resistance. We performed next generation sequencing (NGS) on sequential samples obtained at baseline and after therapy either at PC3, PC5 or PC6. We identified 28 patients in our cohort that did not respond to azacitidine monotherapy and had at least one mutation detected (Table S3). Amongst these 28 patients we observed that 8 had a clonal structure that remained almost unchanged between the two time points, whereas in the remaining 20 we observed changes in the VAFs and/or acquisition of new mutations. Two examples of unchanged clonal structure are depicted in Figure 2A,B and Figure S2A. For patient 70, we sequenced bone marrow (BM) MNCs obtained at diagnosis and post cycle 6 (PC6). At both time points we detected two mutations, namely in *DNMT3A* and *IDH2*. It should be noted that two alternative clonal structures are shown for this patient (Figure 2A, Figure S2A). Imputation from VAFs suggested that *DNMT3A* was acquired first, followed by acquisition of *IDH2*. For this patient we observed that both mutations maintain constant VAFs between the two time points. A similar pattern was observed for patient 16; however, we detected mutation in *SRSF2* and *KRAS* (Figure 2B).

In contrast to the lack of clonal evolution detected in patients 70 and 16, sequencing of samples from patients 123 and 114 revealed intricate patterns of clonal evolution. For patient 123 sequencing was performed on BM MNC samples obtained at baseline and PC3 (Figure 2C). At baseline we detected two independent clones, Clone A with mutations in *DNMT3A* and *MTA2* and Clone B with a mutation in *IDH2*. At PC3, sequencing detected only the mutation in *IDH2* and we observed an increase in the VAF of *IDH2* from 3.7 to 41% suggesting that azacitidine monotherapy favoured growth of Clone B. A complicated example of clonal evolution was detected in patient 114 (Figure 2D). For this patient we sequenced PB MNCs obtained at baseline and BM MNCs obtained at PC5. Sequencing at baseline detected mutations in *IDH2*, *TP53*, *SRSF2*, *NPM1* and *FLT3*. It should be noted that the VAFs of the mutations in *IDH2*, *TP53* and *SRSF2* were very similar and therefore we were unable to determine the order in which mutations were acquired. At PC5 sequencing revealed that the VAFs of *IDH2*, *TP53*, *SRSF2* and *NPM1* remained constant. However, we observed an increase in VAF of *FLT3* from 2.9% to 36%. Additionally, we observed the acquisition of two mutations in *RUNX1*. From our data we concluded that patients with refractory disease have heterogeneous responses to therapy. A similar picture of heterogenous response was also observed in patients that achieved partial response (Table S3).

### 3.3 Clonal basis of response and secondary resistance to azacitidine monotherapy

**FIGURE 3:**
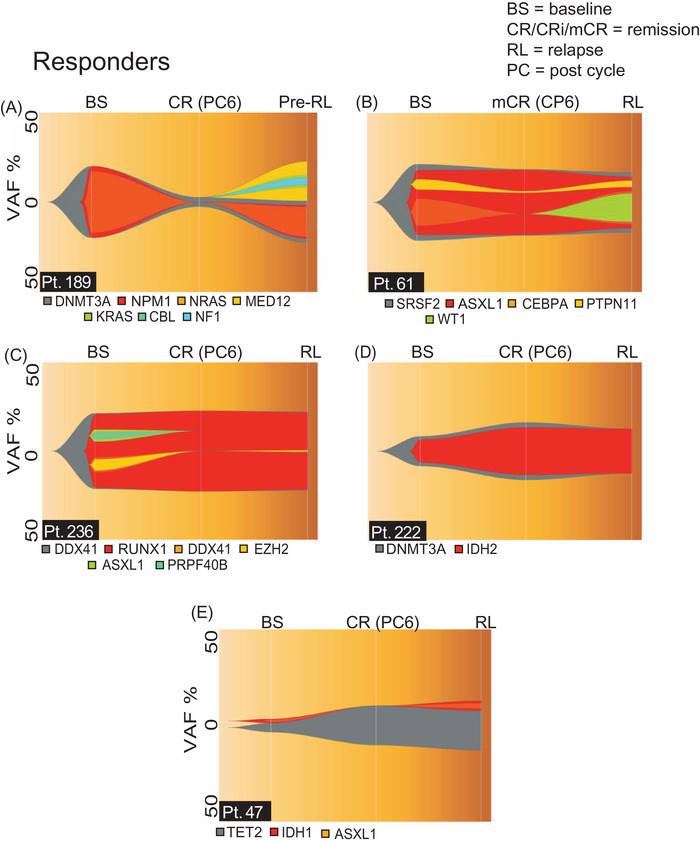
Clonal basis of response and secondary resistance to azacitidine monotherapy. (A–E) FISH plots of patients that responded to therapy, the three time points depicted in the FISH plots represent mutations and VAFs following NGS on samples obtained at baseline (BS), remission (CR, CRi, mCR) and pre‐relapse (Pre‐RL) or relapse (RL). (A) FISH plot of patient 189, (B) FISH plot of patient 61, (C) FISH plot of patient 236, (D) FISH plot of patient 222, (E) FISH plot of patient 47. VAF = variant allele frequency, BS = baseline, CR = complete remission, mCR = marrow CR, Pre‐RL = pre‐relapse, RL = relapse

To study the clonal basis of response to azacitidine monotherapy, we investigated changes in the clonal structure of patients that achieved remission with azacitidine monotherapy. We identified 24 patients that achieved remission and had at least one mutation detected (Table S3). Comparing the mutational burden and VAFs of samples obtained at baseline and remission revealed 3 different types of response: 1) an almost complete elimination of mutations (remaining mutations had VAF <5%) in 33% of the patients (Figure 3A and Figure S3A–G); 2) No differences in the clonal structure between the two time points (17%), (Figure S3H–K); 3) Changes in the clonal structure that vary amongst different patients with no discernible pattern (50%). The third class contained the majority of the patients, including patients 61, 236, 222 and 47 which are representative of certain patterns of clonal evolution (Figure 3B–E). Sequencing of samples obtained at baseline and remission from patients 61 and 236, revealed that some mutations remained unchanged, there was a decrease in the VAFs of others and also some of them were eliminated. In patient 222 we detected mutations in *DNTM3A* and *IDH2* at both time points and we observed that at remission there was a two‐fold increase in the VAFs of both mutations. In patient 47 we detected 3 mutations at baseline in *TET2*, *IDH1* and *ASXL1*. However, the clonal structure changed during remission, as we were only able to detect the mutation in *TET2*. In addition, we observed an increase in the VAF of the *TET2* mutation from 5% to 22%.

Having identified lack of a uniform pattern of response, we turned our attention to investigate changes in the clonal structure at secondary resistance. Towards this end, in addition to sequencing samples obtained at baseline and remission, we also sequenced samples obtained at relapse or pre‐relapse. We observed that in some patients relapse is driven by clonal evolution and in particular the acquisition of new mutations following remission. One such example is patient 189, at baseline we detected 3 mutations, namely in *DNMT3A*, *NPM1* and *NRAS*. Following therapy, at remission, the only mutation detected was in *DNMT3A* with VAF of 5%. However, sequencing of BM MNCs obtained at PC18 revealed the re‐emerge of mutations that were present at baseline and the acquisition of 4 new mutations in *MED12, KRAS*, *CBL* and *NF1* (Figure 3A and Figure S3L shows different clonal structure). Acquisition of a new mutation was also observed for patient 61 (Figure 3B). In patients 189 and 61, we detected dynamic changes in the clonal structure following remission. Although this is only a speculation, for these patients clonal evolution could be the primary cause for secondary resistance. In contrast to these patients, we identified patients with no detectable changes in the clonal structure between remission and relapse (Figure 3C,D). It is clear that for these patients relapse was not driven by clonal evolution. This highlights that other genetic or non‐genetic changes could be involved in secondary resistance to azacitidine monotherapy. From our data we conclude that there is a lack of uniform response and secondary resistance to azacitidine therapy. Additionally, response and secondary resistance could be driven by both genetic and non‐genetic mechanisms.

### 3.4 Different frequencies of cytotoxic and naïve helper T cells between responders and non‐responders are present at baseline

Mutational profiling of sequential patient samples treated with azacitidine revealed a heterogenous response to therapy. Importantly, in some patients we detected changes in the genetic landscape whereas in others we did not. This suggests that response or resistance to azacitidine could be driven by non‐genetic mechanisms. To identify other mechanisms that might play a role in response or resistance, we turned our attention to the immune microenvironment. There have been a number of studies showing that hypomethylating agents trigger an immune mediated anti‐tumour response [17, 18, 19, 20, 21]. For the purposes of this study, we aimed to assess the number and functional states of different immune cell types before and after azacitidine monotherapy in patients that responded to therapy and non‐responders. Towards this end, we established a high‐dimensional immunophenotyping panel (Table S4).

**FIGURE 4:**
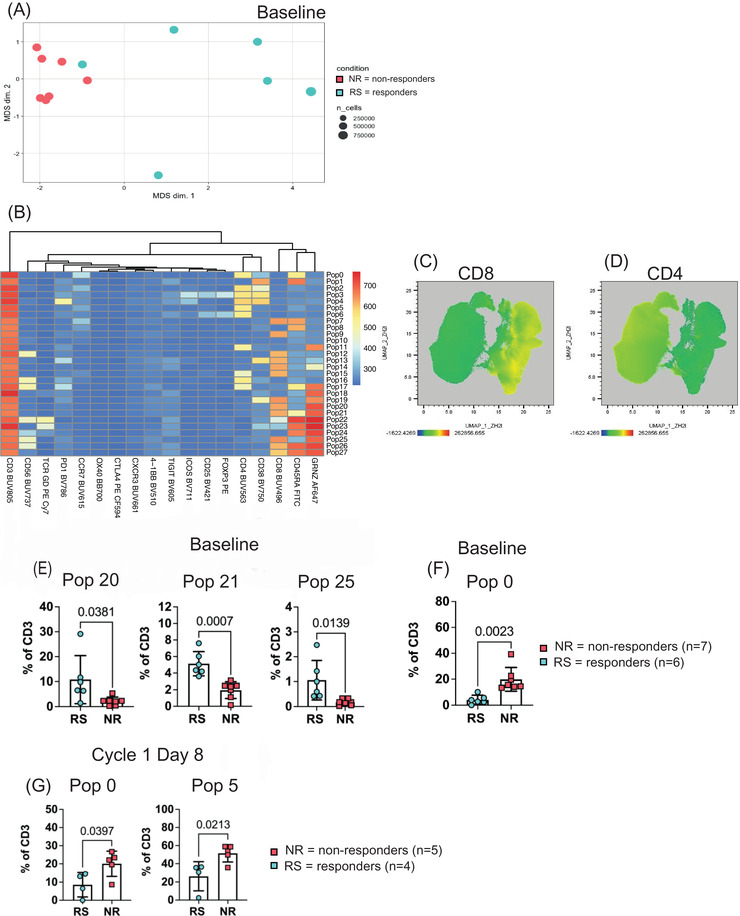
Immunophenotyping of acute myeloid leukaemia (AML) patients before and after azacitidine treatment. (A) Multidimensional scaling plot (MDS) of responders and non‐responders at diagnosis. Each point represents a patient. (B) Heatmap of 28 populations identified with PhenoGraph and FlowSOM. (C and D) UMAPs of CD8 and CD4 expression, respectively. (E) Three populations with higher frequencies detected in responders at baseline. Data are shown as mean ± SD; each dot represents a sample. Student's *t*‐test was performed. (F) One population with higher frequency was detected in non‐responders at baseline. Data are shown as mean ± SD; each dot represents a sample. Student's *t* test was performed. (G) Two populations with higher frequencies in the non‐responders after treatment at cycle 1 day 8. Data are shown as mean ± SD; each dot represents a sample. Student's *t* test was performed

We initially utilised our panel to investigate differences in the immune microenvironment of patients that responded to therapy compared to non‐responders at baseline. Focusing on T cells (Figure S4A), we observed that multidimensional scaling (MDS) plot separated non‐responders and the majority of responders. Additionally, we observed that patients that did not respond to therapy clustered together whereas no clustering was observed for the responders (Figure 4A). In order to identify populations that distinguish responders from non‐responders we generated a concatenated file that contained all the samples (down‐sampled to the smallest sample). We applied recently developed clustering algorithms, PhenoGraph and FlowSOM, which identified 28 phenotypically distinct populations (Figure 4B) [22, 23]. Projection of our data into a uniform manifold approximation and projection (UMAP) revealed separation of the two T cell lineage markers CD8 and CD4 (Figure 4C,D).

Next, we wanted to see if a specific population is more prominent in one group of patients. We observed 4 populations to be differentially represented between responders and non‐responders at baseline. Three populations were higher in the responders, in particular, populations 20, 21 and 25 (Figure 4E and Table 2). Interestingly, these three populations were phenotypically similar, all of them expressing CD8 and GRNZ. However, population 21 also expressed CD45RA and population 25 expressed CD56. Populations 20 and 21 represent phenotypically cytotoxic T cells, whereas, CD56 expression of population 25 suggests that they are unconventional CD8+ NK T cells. The one population that had higher representation in the non‐responders at baseline was population 0 (Figure 4F and Table 2). This population was defined by the expression of CD4, CD38, CD45RA and CCR7 suggesting that this population phenotypically resembles naïve helper T cells (Figure S4B).

**TABLE 2:** Immunophenotype of T cell populations identified to have different frequencies in responders and non‐responders at baseline and cycle 1 day 8 following treatment with azacitidine

Population ID	Immunophenotype
Population 0	CD3+CD4+CD45RA+CCR7+CD38mid/low
Population 5	CD3+CD4+CD45RAlowCCR7+
Population 20	CD3+CD8+GRNZ+
Population 21	CD3+CD8+GRNZ+CD45RA+
Population 25	CD3+CD8+GRNZ+CD56+

### 3.5 Higher frequency of naïve helper T cells persists in non‐responders after therapy

Having identified differences in the immune microenvironment of different groups of patients before azacitidine monotherapy, we also wanted to study the expression of the same molecules after therapy. Towards this end, we performed immunophenotyping using the same panel on samples obtained at cycle 1 day 8, from the same patients. Clustering remained similar for non‐responders, whereas, responders did not cluster (Figure S4C). Next, we compared the frequency of the 28 populations before and after therapy in patients that responded to therapy. We did not detect any uniform changes following therapy. Subsequently, we turned our attention to non‐responders where we observed a decrease in 3 populations of cytotoxic T cells (Figure S4D).

Finally, we wanted to investigate differences between patients that responded to therapy and non‐responders following azacitidine monotherapy at cycle 1 day 8. We identified two populations differentially represented between responders and non‐responders. Both populations had a higher frequency in non‐responders (Figure 4G and Table 2). Population 0 resembles phenotypically naïve helper T cells with low CD38 expression and population 5 also resembles naïve helper T cells but with lower expression of CD45RA. It is interesting, that phenotypically naïve helper T cells appear to have higher frequency in non‐responders before and after therapy.

## 4 DISCUSSION

Azacitidine has been used in the clinic for many years but we still lack understanding of the exact mechanism by which this drug exerts its clinical anti‐tumour effect. We hypothesised that the mutational landscape could play a role in the response, primary resistance and secondary resistance to azacitidine. Targeted sequencing of sequential samples from patients treated with azacitidine did not identify any genetic determinants that could predict response to therapy. In accordance with the literature, we observed that pre‐leukemic mutations persisted following therapy [24, 25]. Additionally, we detected a decrease in the VAF of *DNMT3A* mutations following treatment. This reduction in VAF was present in most patients, suggesting azacitidine might be targeting clones with mutations in *DNMT3A*. Next, we investigated the clonal basis of response, primary and secondary resistance. We detected heterogenous patterns of response; however, we observed that specific patterns of clonal evolution could be used to group the different types of response. Interestingly, focusing on secondary resistance it was clear that in some patients relapse is driven by clonal evolution whereas in others non‐genetic mechanisms might play a role as we did not detect any differences in the clonal structure between remission and relapse. Of course, this could be due to technical limitations associated with using a targeted genotyping panel. Additionally, other genetic mechanisms might be involved e.g. differences in gene expression could also be driving therapy response and resistance.

To identify non‐genetic mechanisms that might play a role, we turned our attention to the immune microenvironment. A number of studies have already shown that azacitidine has an effect on the immune microenvironment. In particular, Goodyear et al. showed that in the post‐transplant setting treatment with azacitidine leads to an increase in the number of Tregs and induction of a cytotoxic T cell response against a number of tumour specific antigens [26]. Additionally, another study showed that treatment with azacitidine resulted in *PD* *1* promoter demethylation in T cells [27]. To further investigate the effect of azacitine on the immune microenvironment we developed a comprehensive immunophenotyping panel that allowed detection of different immune cell types and their functional state. We did not detect any uniform changes in the responders following therapy. However, we did observe higher percentage of cytotoxic T cells in the responders and higher percentage of naïve helper T cells in the non‐responders at baseline. Interestingly, the later remained high in the non‐responders following treatment. This intriguing finding could lead to the speculation that this population could play a role in the lack of response to azacitidine monotherapy. Lamble et al. also describe a high frequency of phenotypically naïve cells in the bone marrow of AML patients that were defined as non‐proliferators [28]. It is intriguing that phenotypically naïve T cells are higher in the non‐responders. Could these cells be critical for the lack of response to azacitidine therapy? Further studies are required to elucidate the role of these cells. Overall, we observed that there is a lack of a uniform response to azacitidine and that response and resistance could be driven by genetic and non‐genetic mechanisms.

## CONFLICT OF INTEREST

PV, CC and VS report receiving research funding from Celgene. The remaining authors report no conflict of interest.

## ETHICS STATEMENT

This study used consented patient samples obtained during the RAvVA trial (ISRCTN68224706, EudraCT 2011‐005207‐32) which was reviewed by an independent research ethics committee in accordance with recognized ethical guidelines. All participants gave written informed consent for use for samples for research in accordance with the Declaration of Helsinki.

## AUTHOR CONTRIBUTIONS

V.S. designed and performed experiments, analysed data and wrote the manuscript. M.M. performed experiments and analysed data. B.U. collected and processed patient samples. A. H. and S.F. provided clinical data. C.C. provided clinical data. P.V. conceived and supervised the study.

## Supporting information

Supplementary Figures

Table S1 Information

Table S2 Information

Table S3 Information

Table S4 Information

## Data Availability

The data that support the findings of this study are available from the corresponding author upon reasonable request.
