# Supplementary material for: Heterogeneous genetic and non‐genetic mechanisms contribute to response and resistance to azacitidine monotherapy
Source: EJHaem. 2022 Jul 8;3(3):794–803. doi: 10.1002/jha2.527 (PMC9421974; doi:10.1002/jha2.527)
Supplement: Supplementary file 1 — Supplementary Figures [file JHA2-3-794-s002.docx]

**Materials and methods**

**Next Generation Sequencing and bioinformatic analysis**

DNA was extracted from BM and PB MNCs using DNeasy Blood and Tissue kit (Qiagen) according to manufacturer’s instruction.

Targeted DNA sequencing was performed on BM and PB MNCs DNA samples. Libraries were prepared using the KAPA HyperPlus (Roche) protocol according to manufacturer’s instructions, starting with 100ng of DNA and adding 6 PCR cycles. Targeted capture was performed on these libraries using a custom pool of biotinylated capture probes (SeqCap EZ Prime Choice, Roche) targeting 108 genes recurrently mutated in myeloid malignancies. Amplified DNA libraries were hybridized to the capture probes in pools of 12 samples according to the manufacturer’s instructions. Libraries were pooled in equimolar concentrations and were sequenced on an Illumina NextSeq 500 using 75 paired-end reads.

The pipeline used for variant calling is described in Korber *et al*. ^1^ For the purposes of this study, in addition to the exclusion/inclusion criteria described in Korber *et al*, we only included variants that were called by both VarDict^2^ and Mutect2^3^, except for FLT3_IDT which was called by Pindel^4^. Variants were considered germline if the VAF was between 40-60% and constant between sequential samples unless it has previously been reported in COSMIC. We included variants with a minimum VAF of 2% or 1-2% if they were reported in another time point with higher VAF.

We used the package fishplot to visualise tumour evolution.^5^

**Droplet digital PCR**

Droplet digital PCR was utilized for calculation of VAF of G646Wfs*12 mutation in ASXL1 for patient 148. We used the Bio-Rad platform starting with 40ng of gDNA and used primers Frw: CCTCGCAGACATTAAAGCCC and Rev: CACCAC CATCACCAC TGC TG and dual labelled hydrolysis probes ASXL1_G646fsins_WT /5’HEX/GGCCACCCCCCCCTC CGA TG, ASXL1_G646fsins_MUT 5’6/FAM/GGC CAC CCCCCCCCTCCG ATG. Fluorescence of droplets was analyzed using a 2-color detector (FAM: wild-type/hexachloro-fluorescein: mutant). The VAFs were used in Supplementary Figure 3A.

**Immunophenotyping**

Frozen PB MNCs from AML and MDS patients were thawed and washed with Iscove’s Modified Dulbecco’s Medium (Thermo Fisher Scientific), 10% fetal bovine serum (Sigma) and 1mg/ml bovine pancreatic DNAse I (Sigma).

List of antibodies used: CD16 BUV395 (3G8), CD8 BUV496 (SK1), CD4 BUV563 (DK3), CCR7 BUV615 (2-L1-A), CXCR3 BUV661 (1C6.CXCR3), CD56 BUV737 (NCAM16.2), CD3 BUV805 (SK7), CD25 BV421 (M-A251), CD137 BV510 (4B4-1), TIGIT BV605 (A15153G), CD278 BV711 ( DX29), CD38 BV750 (HIT2), CD279 BV786 (EH12.1), CD45RA FITC(HI100), CD134 BB700 (ACT35), FOXP3 PE (259D/C7), CTLA-4-PE CF594 ( BNI3),TCRγδ PE-CY7 (B1), Granzyme-B AF467 (GB11), CD14 APC-Cy7 (63D3), CD19 APC-Cy7 (HIB19), CD33 APC-Cy7 (WM53), CD34 APC-Cy7 (581), CD123 APC-F750 (6H6).

Initially, samples were stained with Live/Dead™ fixable dead cell Stainer (Thermo Fisher Scientific) for 20 min at 4°. Next, cell surface markers were added and incubated for 20 min at 4°. For the intracellular staining the Transcription Factor Buffer Set (BD) was used. Fixing and permealisation was performed in accordance to manufacturer’s instruction. Analysis was carried out on BD FACSymphony A5 cell analyser.

Analysis of immunophenotyping data was performed using R studio (Version 1.3.1073) and FlowJo (Version 10.8).

To analyse immunophenotyping data, we initially performed clean-up of the data by gating on cells🡪 singlets 🡪 live, lin-blasts- cells🡪 CD3+ cells for the T cells. We next imported these data into R studio, followed by analysis with the catalyst package^6^.

In FlowJo, samples were initially downsampled and normalized to the smallest sample. The samples were then concatenated into one fcs file followed by PhenoGraph^7^ to get the number of clusters. The output of PhenoGraph was inputted in FlowSOM^8^ and the populations referred in our manuscript are the output of FlowSOM. We used UMAPs for visualization.^9^

**Statistical analysis**

GraphPad Prism (version 9.2.0) was used for statistical analysis. The specific statistical tests performed are indicated in the figure legends.

**Supplementary Figures**

A


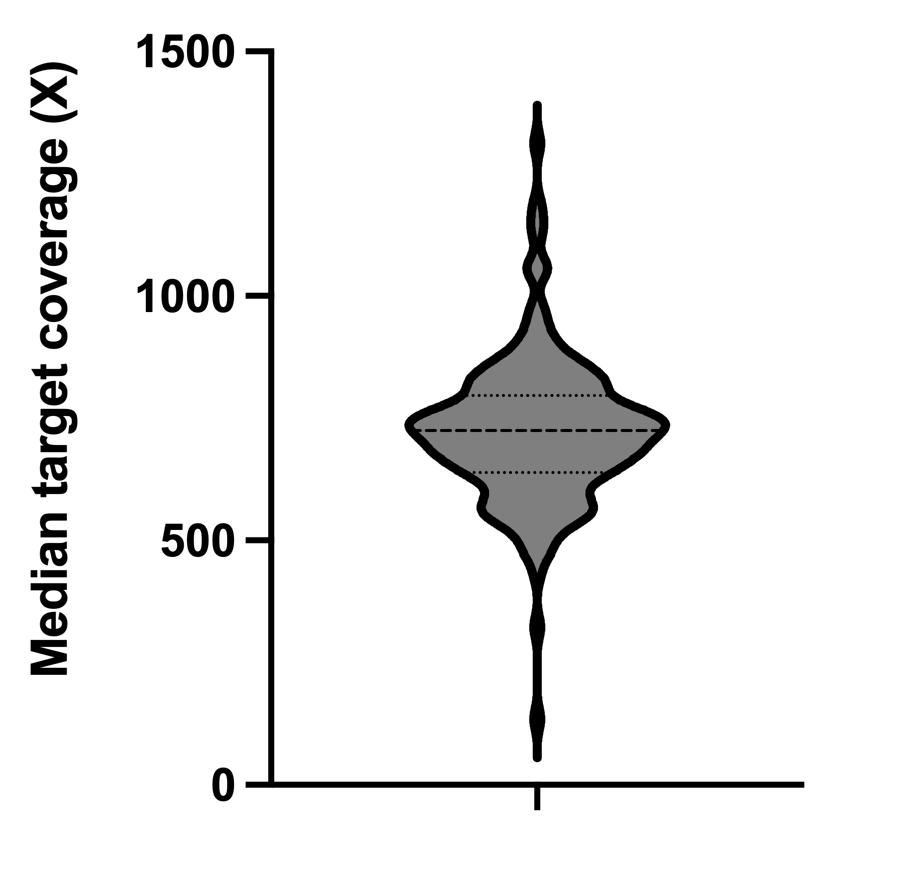


B


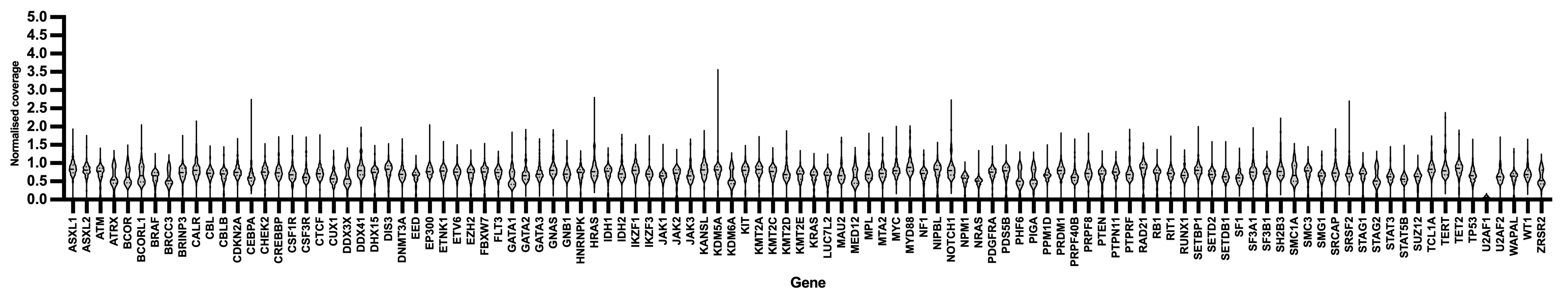


Responders

C


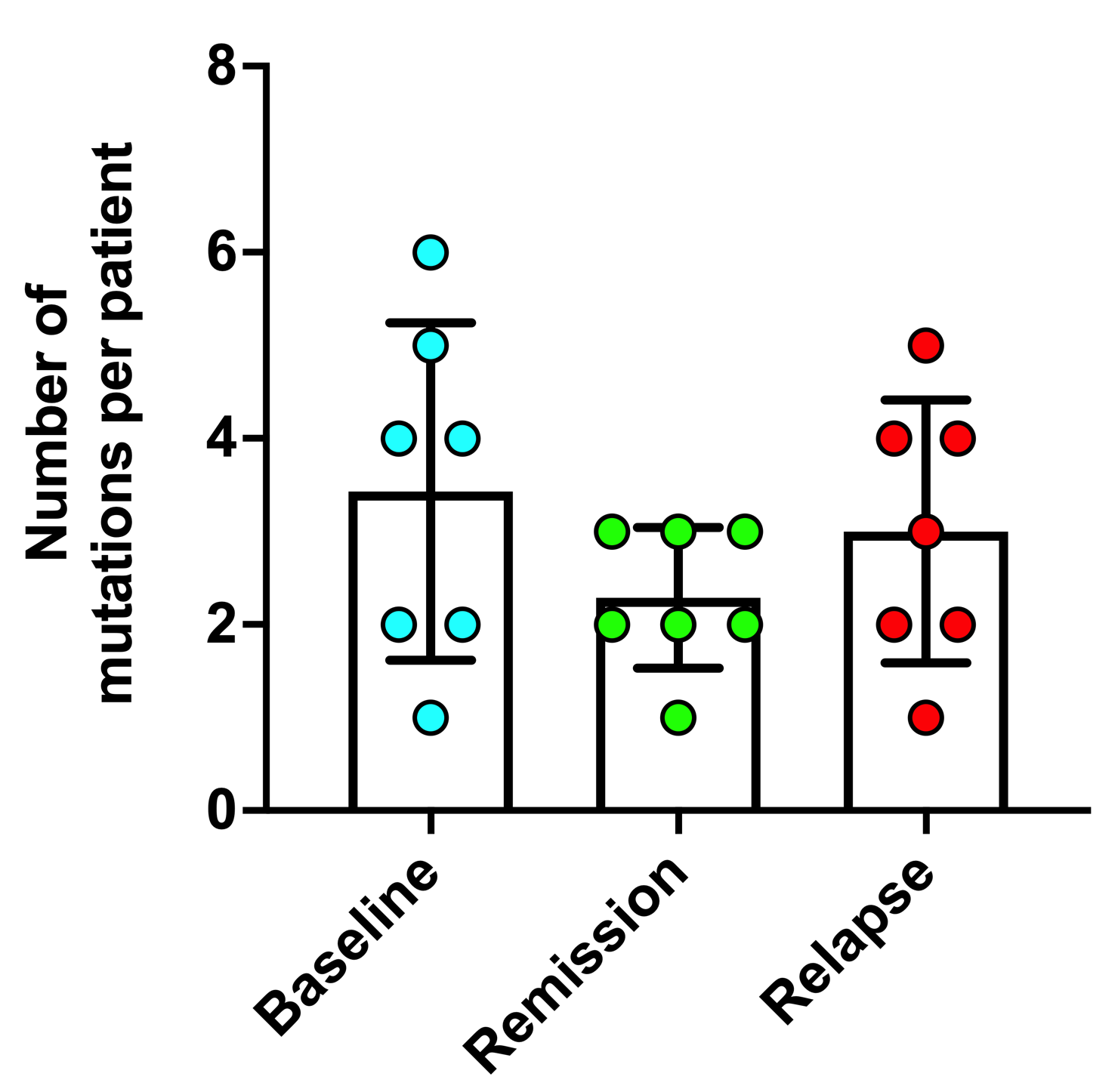


D

Partial-responders

E

Partial-responders


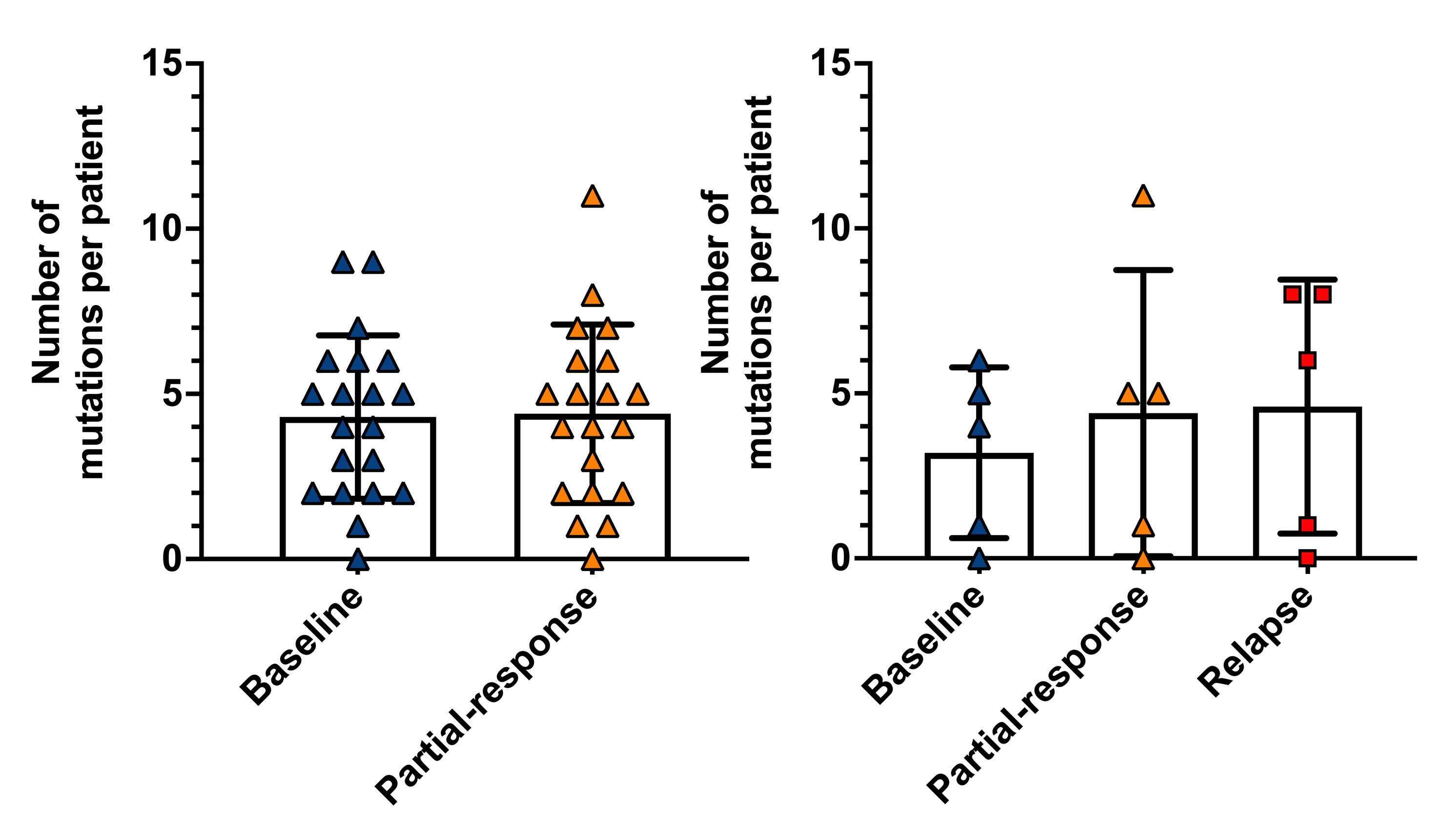

F

Partial-responders


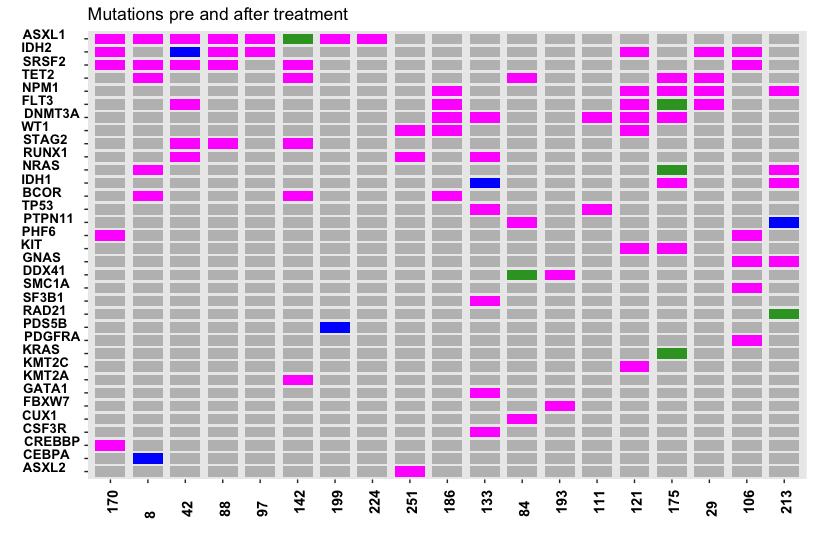


Ho

G

Remission

Baseline


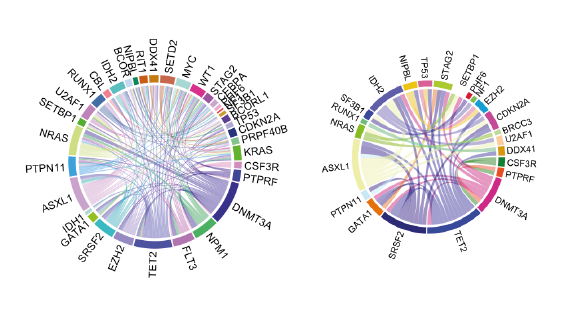


**Supplementary Figure 1| Delineation of the mutational profile of patients before and after azacitidine treatment.**

1. Median coverage across all samples. B) Normalised coverage across each gene in the targeted panel. Low coverage of U2AF1 was due to the fact that in hg38, there is a duplication of the U2AF1 gene on chromosome 21 called U2AF1L5. C) Number of mutations in responders at baseline, remission and relapse. Each point represents a patient. ANOVA test was performed D) Number of mutations in partial-responders at baseline and partial-response. Each point represents a patient. ANOVA test was performed. E) Number of mutations in partial- responders at baseline, partial-response and relapse. Each point represents a patient. ANOVA test was performed. F) Mutations present before and after treatment in partial-responders. Blue = mutations present only before treatment, Green = mutations present only after treatment, Magenta = mutations present at both time points. The y-axis shows the genes where the mutation is present and the x-axis the individual patient. Circos plots of responders at G) baseline and H) remission (n=22). The length of the arch is indicative of the frequency of the mutations and the width of the ribbon is indicative of the frequency of the two mutations co-occurring.

A

**Supplementary Figure 2| Clonal basis of primary resistance to azacitidine monotherapy**

A) FISH plot of patient 70 (alternative clonal structure). Mutations and Variant Allele Frequencies (VAFs) are shown at baseline (BS) and post-cycle 6.


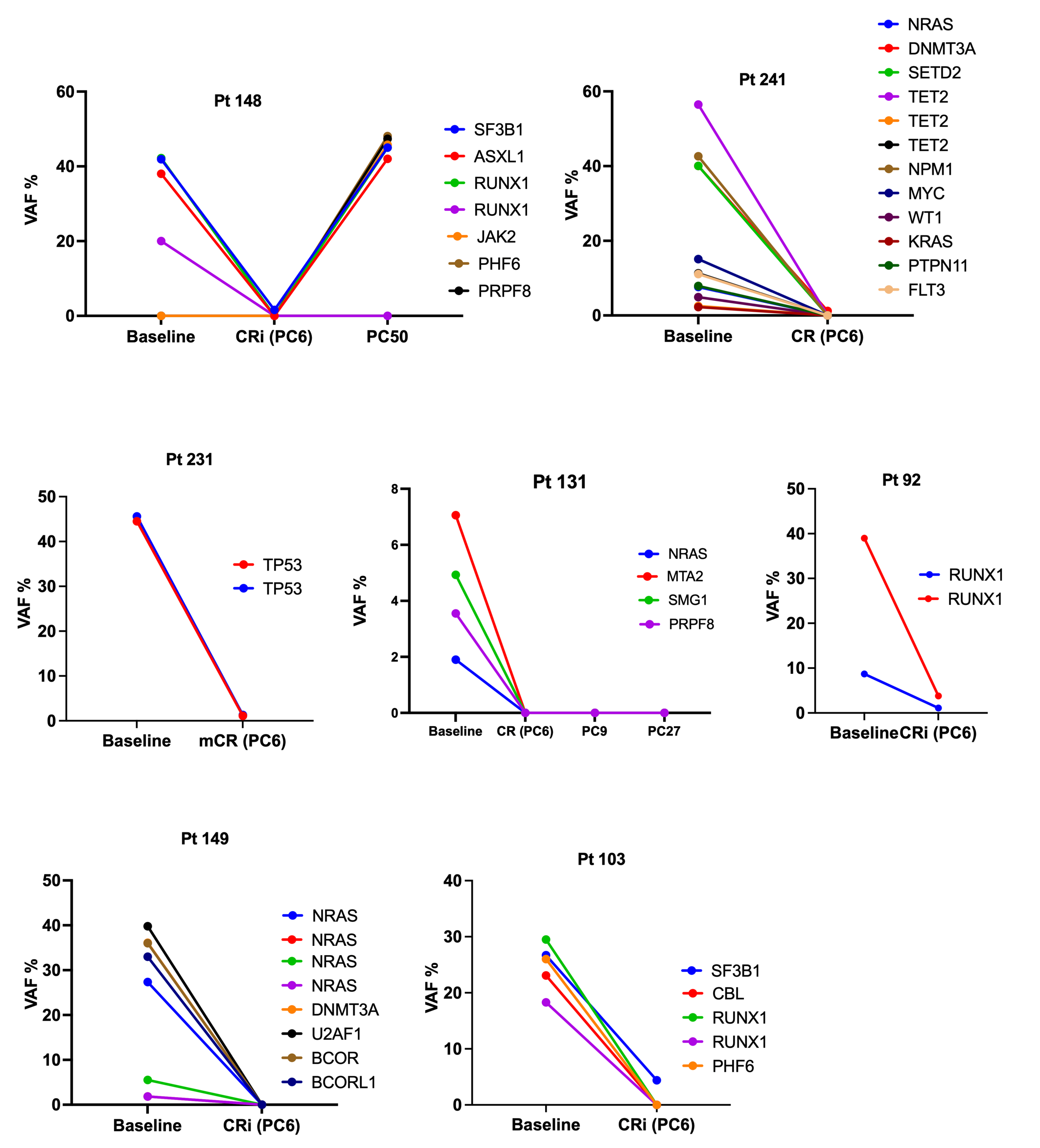


C

D

G

F

E

B

A


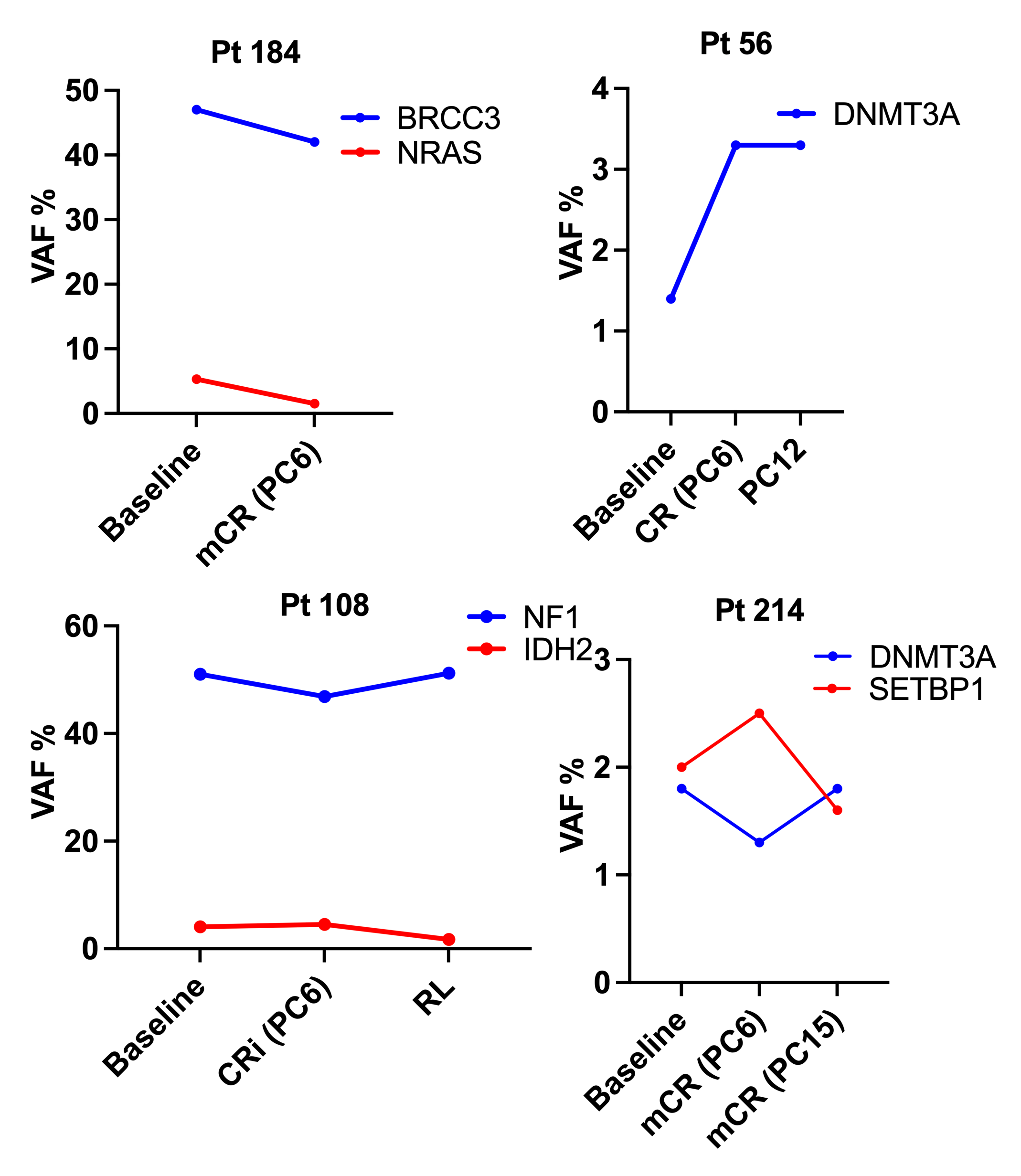


I

K

J

H

L

**Supplementary Figure 3| Clonal basis of response and secondary resistance to azacitidine monotherapy**

A-K) Mutations and Variant Allele Frequencies (VAFs) of responders at different time points for different patients. L) FISH plot of patient 189 (alternative clonal structure). CR= complete remission, CRi CR with incomplete haematological recovery, mCR=Marrow CR, PC=post cycle, RL=relapse, Pre-RL=pre-relapse

A

B

D

C


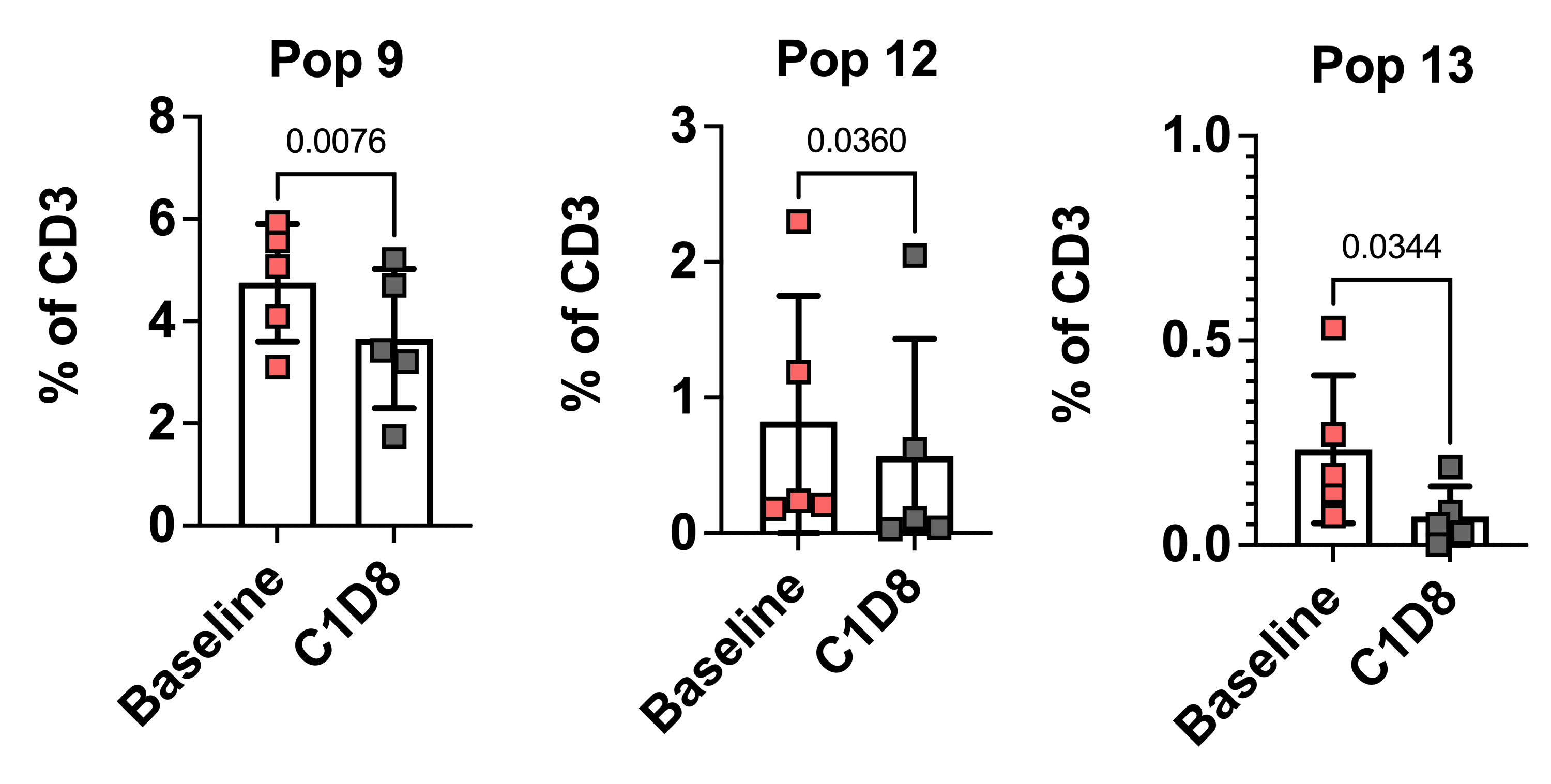


**Supplementary Figure 4|Immunophenotyping of responders and non-responders before and after azacitidine treatment**.

A) Gating strategy for unsupervised analysis of T cells. B) Representative FACS plots of naïve T helper cells from one patient that did not respond to therapy and a responder. C) Multidimensional scaling plot (MDS) of responders and non-responders after therapy at cycle 1 day 8. D) Decrease in the frequency of 3 populations following azacitidine therapy only in non-responders. Data are shown as mean $\pm$SD, each dot represents a sample. Paired-Student’s t test was performed.

**References for materials and methods**

1 Verena Körber , N. A.-P., Niels Asger Jakobsen , Rachel Moore , Nina Claudino , Marlen Metzner , Batchimeg Usukhbayar , Mirian Angulo Salazar , Simon Newman , Benjamin JL Kendrick , Adrian H Taylor , Rasheed Afinowi-Luitz , Roger Gundle , Bridget Watkins , Kim Wheway , Debra Beazley , Andrew J Carr , Paresh Vyas , Thomas Höfer.A simple and direct method to define clonal election in somatic mosaicism (BioRxiv, 2021). https://doi.org/10.1101/2021.12.15.472780

2 Lai, Z. *et al.* VarDict: a novel and versatile variant caller for next-generation sequencing in cancer research. *Nucleic Acids Res* **44**, e108 (2016). <https://doi.org:10.1093/nar/gkw227>

3 Benjamin*, D. & , T. S., Kristian Cibulskis, Gad Getz, Chip Stewart and Lee Lichtenstein. (bioRxiv, 2019).

4 Ye, K., Schulz, M. H., Long, Q., Apweiler, R. & Ning, Z. Pindel: a pattern growth approach to detect break points of large deletions and medium sized insertions from paired-end short reads. *Bioinformatics* **25**, 2865-2871 (2009). <https://doi.org:10.1093/bioinformatics/btp394>

5 Abelson, S. *et al.* Prediction of acute myeloid leukaemia risk in healthy individuals. *Nature* **559**, 400-404 (2018). <https://doi.org:10.1038/s41586-018-0317-6>

6 Nowicka, M. *et al.* CyTOF workflow: differential discovery in high-throughput high-dimensional cytometry datasets. *F1000Res* **6**, 748 (2017). <https://doi.org:10.12688/f1000research.11622.3>

7 Levine, J. H. *et al.* Data-Driven Phenotypic Dissection of AML Reveals Progenitor-like Cells that Correlate with Prognosis. *Cell* **162**, 184-197 (2015). <https://doi.org:10.1016/j.cell.2015.05.047>

8 Van Gassen, S. *et al.* FlowSOM: Using self-organizing maps for visualization and interpretation of cytometry data. *Cytometry A* **87**, 636-645 (2015). <https://doi.org:10.1002/cyto.a.22625>

9 Leland McInnes , J. H., James Melville. UMAP: Uniform Manifold Approximation and Projection for Dimension Reduction (arxiv, 2018). https://doi.org/10.48550/arXiv.1802.03426
